# Activation of the NLRP3/caspase-1 inflammasome in human dental pulp tissue and human dental pulp fibroblasts

**DOI:** 10.1007/s00441-015-2118-7

**Published:** 2015-02-17

**Authors:** Wenkai Jiang, Haipeng Lv, Haijing Wang, Diya Wang, Shukai Sun, Qian Jia, Peina Wang, Bing Song, Longxing Ni

**Affiliations:** 1 State Key Laboratory of Military Stomatology, Department of Operative Dentistry & Endodontics, School of Stomatology, The Fourth Military Medical University, 710032 Shaanxi, People’s Republic of China; 2 Department of Occupational & Environmental Health and the Ministry of Education Key Lab of Hazard Assessment and Control in Special Operational Environment, School of Public Health, Fourth Military Medical University, Xi’an, People’s Republic of China; 3 Tissue Engineering and Regenerative Dentistry, School of Dentistry, Cardiff University, Heath Park, Cardiff, CF14 4XY UK

**Keywords:** Innate immune system, Inflammasome, NLRP3, Dental pulp, Fibroblasts, Human

## Abstract

The NLRP3/caspase-1 inflammasome pathway plays an important role in cellular immune defence against bacterial infection; however, its function in human dental pulp tissue and human dental pulp fibroblasts remains poorly understood. We demonstrate that NLRP3 protein expression occurs to a greater extent in pulp tissue with irreversible pulpitis than in normal pulp tissue and in tissue with reversible pulpitis. Caspase-1 is present in its active (cleaved) form only in pulp tissue with irreversible pulpitis. NLRP3 and caspase-1 are expressed in the odontoblast layers in normal human dental pulp tissue, whereas in inflamed pulp tissue, the odontoblast layers are disrupted and dental pulp cells are positive for NLRP3 and caspase-1. Additionally, we investigate the role of the NLRP3/caspase-1 inflammasome pathway in human dental pulp fibroblasts and show that ATP activates the P2X_7_ receptor on the cell membrane triggering K^+^ efflux and inducing the gradual recruitment of the membrane pore pannexin-1. Extracellular lipopolysaccharide is able to penetrate the cytosol and activate NLRP3. Furthermore, the low intracellular K^+^ concentration in the cytosol triggers reactive oxygen species generation, which also induces the NLRP3 inflammasome. Thus, the NLRP3/caspase-1 pathway has a biological role in the innate immune response mounted by human dental pulp fibroblasts.

## Introduction

Dental caries is a chronic infectious disease. Infection by caries-related bacteria is the main cause of dental caries, which can lead to the demineralization of enamel and dentin and subsequent pulp tissue injury (Akira et al. 2006; Bolwell 1999). Pulpitis is a common inflammatory disease that occurs in the pulpal connective tissues when the infection of caries-related microorganisms reaches the dentinal tubules and pulp (Bortoluci and Medzhitov 2010). The bacterial infection that causes the advance of dental caries is reported to initiate an immune response (Botero et al. 2006). The innate immune system components in the pulp are the first line of defence against bacterial infection of the pulp of carious teeth (Chang et al. 1998). Two main types of mesenchymal cells are found in human dental pulp tissue, namely odontoblasts and fibroblasts, which differ in both location and function (Cooper et al. 2010). Odontoblasts are located at the periphery of the pulp and are the first cells to encounter a bacterial infection. These cells express a number of Toll-like receptors (TLRs), including TLR4 and TLR9 and release chemokines, including interleukin-8 (IL-8) and IL-1β, upon the recognition of carious bacteria and/or bacterial products (Cooper et al. 2010; Dostert et al. 2008; Durand et al. 2006; Fay et al. 2006; Feldmeyer et al. 2007; Franchi et al. 2012). In contrast, human dental pulp fibroblasts (HDPFs), the main cells that constitute the dental pulp, produce pro-inflammatory factors (which are involved in the innate immune response) when stimulated by caries-related bacterial virulence factors. TLR, nucleotide-binding oligomerization domain containing 1 (NOD1) and NOD2 expression has been detected in HDPFs (Guarda et al. 2011). Taken together, these findings suggest that odontoblasts and HDPFs play a role in the innate immune response and thereby protect against bacterial infection in the dental pulp.

Pattern recognition receptors (PRRs) are expressed by many cells including macrophages, monocytes, dendritic cells, neutrophils and epithelial cells. An understanding of the function of PRRs has provided great insights into host-mediated innate immune responses to microbial attack. These cells can “scan” the extracellular milieu and endosomal compartments for pathogen-associated molecular patterns (PAMPs; Hahn et al. 2000; Harder et al. 2009). One category of intracellular PRRs, namely NOD-like receptors (NLRs), recognizes PAMPs and host-derived danger signals (danger-associated molecular patterns) and plays an important role in the innate immune response (Hahn et al. 2000; He et al. 2010). The NLRs, which are key components of inflammasomes, are encoded by 22 genes in humans and by many more genes in mouse (Hahn et al. 2000; Hirao et al. 2009). The NLRP3 (NACHT, LRR [leucine-rich repeat] and PYD [pyrin domain] domains-containing protein 3) inflammasome has emerged as the most versatile innate immune receptor because of its broad specificity; this inflammasome mediates the immune response to a wide range of microbial or danger signals (Hirao et al. 2009). NLRP3 mediates the assembly of the inflammasome complex in the presence of microbial components, leading to the activation of caspase-1 and the processing and release of the pro-inflammatory cytokines IL-1β and IL-18 (Hahn et al. 2000; Hirao et al. 2009). NLRP3 can oligomerize and recruit the adaptor protein ASC (apoptosis-associated speck-like protein containing a caspase recruitment domain [CARD]) by using pyrin-domain interactions. Subsequently, pro-caspase-1 is recruited by ASC through CARD-CARD interactions, which leads to the formation of the NLRP3 inflammasome and caspase-1 activation (Hahn et al. 2000; Hoshino 1985). Caspase-1 is synthesized as a 45-kDa pro-caspase (p45), which upon cleavage generates two proteins of 10 and 20 kDa (p10 and p20) that form a hetero-tetrameric complex with enzymatic activity (Kahlenberg and Dubyak 2004; Kanneganti et al. 2007). The appearance of p20 and p10 in culture supernatants therefore reflects caspase-1 activation (Kahlenberg and Dubyak 2004; Anand et al. 2011). Some studies have reported that immune cells, e.g., macrophages that are stimulated by bacterial virulence factors such as lipopolysaccharide (LPS), can activate the NLRP3 inflammasome pathway and the release of the pro-inflammatory factor IL-1β, which is involved in innate immune responses. However, whether non-immune cells are involved in the innate immune response through the NLRP3/caspase-1 inflammasome pathway is unclear.

The NLRP3 inflammasome can be activated in a number of ways; one way is through the action of adenosine triphosphate (ATP) on the P2X_7_ receptor. The purinergic P2X_7_ ATP-gated ion channel can be activated by extracellular ATP (Kawai and Akira 2011), thus triggering K^+^ efflux and inducing the gradual recruitment of the pannexin-1 membrane pore (Keller et al. 2008). Pore formation allows extracellular NLRP3 agonists to access the cytosol and directly activate NLRP3 (Keller et al. 2008). The production of reactive oxygen species (ROS), another activator of NLRP3, is potentially correlated with the K^+^ concentration in the cytosol (Kokkas et al. 2007). Previous studies have focused on the role of purinergic P2X_7_ ATP-gated ion channels and ROS in immune cells; however, whether the P2X_7_ ATP-gated ion channels and ROS production also function in non-immune cells remains unclear.

The purpose of this study is to elucidate the expression, location, distribution and concentration of the NLRP3/caspase-1 inflammasome in human dental pulp tissue. Additionally, we examine the expression of ATP-gated ion channels in HDPFs in vitro and the ability of LPS to activate the NLRP3/caspase-1 inflammasome pathway and to cause the release of the inflammatory cytokine IL-1β. We thus provide a basis for further investigation of the role of NLRP3/caspase-1 in the innate immune response.

## Materials and methods

### Sample collection and preparation

The subjects in this study were recruited from the Department of Oral and Maxillofacial Surgery at the School of Stomatology, Fourth Military Medical University, Xi’an, China. Twenty-seven human third molars, including nine free from caries, nine carious teeth with irreversible pulpitis and nine carious teeth with reversible pulpitis, were collected for the preparation of dental pulp specimens, as described previously (Kummer et al. 2007). Clinically, teeth with irreversible pulpitis are sensitive to heat and have spontaneous lingering pain and teeth with reversible pulpitis show instant pain in cold sensitivity tests. Caries-free teeth from volunteers who had no clinical medical history and who were taking no medications were collected as controls. Clinical and radiographic examinations were used to exclude teeth with a diagnosis of pulp necrosis, periapical pathosis, periodontal diseases or other injuries, with the exception of crown fractures or any restoration in normal dental pulp. Written informed consent was obtained from all volunteers and routine surgical procedures were used. The ethics committee of the Fourth Military Medical University School of Stomatology approved the experimental protocols (permission number IRB-REV-2012-017). The specimens were used for real-time quantitative reverse-transcriptase polymerase chain reaction (qRT-PCR; *n* = 3), Western blot analysis (*n* = 3) and immunohistochemical staining (*n* = 3).

The tooth samples (three in each group) used for histological examination were immediately fixed in 4 % paraformaldehyde for 24 h at room temperature after the root tips had been removed (3 mm to the apical). The specimens were further demineralized in 17 % ethylenediamine tetra-acetic acid solution (pH 6.4) for roughly 3 months. The teeth were processed for paraffin embedding after complete demineralization. Serial tissue sections (5 μm thickness) were collected on silane-coated glass slides and prepared for immunofluorescence staining.

### Cell culture and chemical reagents

Impacted third molars were collected from adults (18–22 years old) with the patient’s informed consent at the School of Stomatology, the Fourth Military Medical University. The ethics committee of the Fourth Military Medical University School of Stomatology approved the experimental protocols (permission number IRB-REV-2012-017). HDPFs were cultured and characterized as previously reported (Mariathasan and Monack 2007). Briefly, impacted third molars were extracted and the root was immediately removed by horizontal section below the cemento-enamel junction with a number 330 bur at high speed with a water spray. The pulp tissue was removed aseptically, rinsed with phosphate-buffered saline (PBS without serum) and placed in a 10-mm glass Petri dish. Pulp tissue was cut into small fragments by using surgical blades and the tissue fragments were seeded onto the plastic surface of a T_25_ tissue culture flask by using a sterile needle. The alpha modification of Eagle’s medium (α-MEM) supplemented with 20 % fetal bovine serum (FBS), 100 IU/ml penicillin, 100 g/ml streptomycin and 2 mM glutamine (Gibco, USA) was used as the culture medium (200 μl). Cultures were maintained at 37 °C in a humidified atmosphere of 5 % CO_2_. The volume of the medium was increased by 500 μl every other day. When the cell colonies reached approximately 80 % confluence, the cells were collected to isolate single cell clones by limiting dilution. The cells from these clones were observed and passage 0 (P0) cells were cultured and expanded for the following experiments. Cells at the third or fourth passages were used for this study. ATP, LPS (from *Escherichia coli* serotype O111:B4), N-acetyl-L-cysteine (NAC), potassium chloride (KCl) and dimethyl sulfoxide (DMSO) were purchased from Sigma-Aldrich (USA). Glibenclamide (Gliben) was purchased from Enzo Life Sciences (USA).

### Collection of human pulp tissue and HDPFs

Dental pulp tissue was obtained by using a previously described method and was examined at the gene and protein level (Martin et al. 2002). Briefly, the pulp tissue was collected from extracted teeth with a number 330 bur at high speed with a water spray and then placed on ice and stored at −70 °C. HDPFs at the fourth passage were isolated as described above and were dissolved in RNA plus (Takara, Otsu, Japan) for qRT-PCR analysis or in lysis buffer (Santa Cruz Biotechnology, Calif., USA) for Western blotting and enzyme-linked immunosorbent assay (ELISA) analysis.

### Analysis by qRT-PCR

Total RNA was extracted from pulp tissue and HDPFs by using RNA plus (Takara) and treated with RNase-free DNase I (RQ1; Promega, Madison, Wis., USA). One microgram of total RNA was used as a template to make first-strand complementary DNA by oligo-dT priming with the Omniscript RT kit (Qiagen, Valencia, Calif., USA). qRT-PCR analyses were performed in an ABI Prism 7500 Real-Time PCR System (Applied Biosystems, Foster City, Calif., USA) with the SYBR Green PCR master mix reagent (Takara) in a 40-cycle PCR. The denaturing, annealing and extension conditions of each PCR cycle were 95 °C for 30 s, 95 °C for 5 s, 60 °C for 34 s and 95 °C for 15 s. The relative amount or fold change of the target gene expression was normalized relative to the level of D-glyceraldehyde-3-phosphate dehydrogenase and relative to a control (untreated cells). The primer sequences used in the qRT-PCR were as follows:

**Table:** 

Genes	Primers	Sequences	Accession number
GAPDH (D-glyceraldehyde-3-phosphate dehydrogenase)	Forward primer	5′-GCACCGTCAAGGCTGAGAAC-3′	NM_002046.3
	Reverse primer	5′-TGGTGAAGACGCCAGTGGA-3′	
P2X_7_	Forward primer	5′-CACTCGGATCCAGAGCATGAA-3′	NM_002562.5
	Reverse primer	5′-CAGCTTGTCACTCACCAGAGCA-3′	
NLRP3 (NACHT, LRR and PYD domains-containing protein 3)	Forward primer	5′-GATCTTCGCTGCGATCAACA-3′	NM_001127461.2
	Reverse primer	5′-GGGATTCGAAACACGTGCATTA-3′	
Caspase-1	Forward primer	5′-GCCTGTTCCTGTGATGTGGAG-3′	NM_033295.2
	Reverse primer	5′-TGCCCACAGACATTCATACAGTTTC-3′	
IL-1β (interleukin-1β)	Forward primer	5′-CCAGGGACAGGATATGGAGCA-3′	NM_000576.2
	Reverse primer	5′- TTCAACACGCAGGACAGGTACAG-3′	

### Western blot analysis

The total protein content was extracted from pulp tissue and HDPFs by using lysis buffer containing protease inhibitors (Sigma-Aldrich, USA). The protein concentration was measured by using a BCA-200 protein assay kit (Pierce, Rockford, Ill., USA). Equal amounts of protein were separated by sodium dodecyl sulfate/polyacrylamide gel electrophoresis and transferred to a polyvinylidene fluoride membrane. The membrane was blocked in TRIS-buffered saline with Tween (TBST) containing 5 % nonfat dry milk for 2 h. For the detection of caspase-1, the membranes were probed overnight at 4 °C with a rabbit anti-human caspase-1 antibody diluted 1:100 (Abcam, US) and then incubated for 2 h with a horseradish-peroxidase-conjugated anti-rabbit IgG antibody diluted 1:10,000 (Santa Cruz Biotechnology, Calif., USA). For the detection of NLRP3, the membranes were incubated overnight at 4 °C with a rabbit anti-human NLRP3 antibody diluted 1:100 (Abcam, USA) and then incubated for 2 h with a horseradish-peroxidase-conjugated anti-rabbit IgG antibody diluted 1:10,000 (Santa Cruz Biotechnology). Protein bands were visualized on X-ray film by using an enhance chemiluminescence system (GE Healthcare, Buckinghamshire, UK). The relative protein expression intensities were quantified by densitometry by using Quantity One analysis software.

### ELISA analysis

The HDPFs were counted and plated on 6-well plates (1 × 10^6^ cells per well) in three wells per group. After 24 h, α-MEM was replaced with various media, as described below. Cells were exposed to ATP (5 mM) for 2 h and then pretreated with specific inhibitors for the indicated time, followed by LPS exposure at 10 μg/ml for 6 or 12 h. After incubation, the media were removed and the cells were washed with PBS at 4 °C. During protein extraction, the plates were kept on ice to avoid the denaturation of the cytokines. Each well was filled with 100 μl lysis buffer, supplemented with 10 μl protease inhibitor (Sigma, US) for 15 min; subsequently, the cells were collected by vigorous scraping by using a cell scraper, transferred into Eppendorf centrifuge tubes and centrifuged at 4 °C at 12,000*g* for 15 min. The supernatant was collected after centrifugation. The amount of IL-1β protein in the culture medium was quantified by using a commercially available human-specific ELISA kit, following the manufacturer’s instructions (eBioscience, San Diego, Calif., USA).

### Immunofluorescence staining and flow cytometry

HDPFs at the fourth passage were probed with a mouse monoclonal anti-vimentin antibody diluted 1:150 (Boster, China), a rabbit monoclonal anti-keratin diluted 1:150 (Boster), a rabbit monoclonal anti-NLRP3 antibody diluted 1:100 and a rabbit monoclonal anti-caspase-1 antibody diluted 1:100. Three randomly chosen 5-μm-thick sections were probed with a rabbit monoclonal anti-NLRP3 antibody (1:100) and a rabbit monoclonal anti-caspase-1 antibody (1:100). Cells were fixed with 4 % paraformaldehyde for 30 min and then incubated in PBS containing 0.4 % Triton X-100 for 10 min on ice. The sections were treated with 3 % H_2_O_2_ for 20 min to block the endogenous peroxidase after having been dewaxed and dehydrated. The sections were then immersed in sodium citrate buffer (0.01 mol/l; pH 6.0) for 20 min at 95 °C for antigen retrieval. The cells and sections were blocked with bovine serum albumin (BSA) for 60 min at 37 °C. After the blocking step, the cells and sections were incubated with primary antibody at 4 °C overnight; PBS was used as the negative control. The cells and sections were then washed with PBS and incubated for 1 h with the secondary antibodies, namely anti-rabbit IgG conjugated to fluorescein isothiocyanate (FITC) or anti-mouse IgG conjugated to rhodamine (Jackson ImmunoResearch, West Grove, Pa., USA), each at 1:1000, at room temperature. After being rinsed with PBS, the sections and cells were examined under an Olympus optical microscope or a LASER confocal microscope (Olympus, Japan).

The percentages of CD29-, CD90-, CD34-, CD45-, CD146 - and STRO-1- positive cells were analysed by flow cytometry. HDPFs at the third passage (>2 × 10^5^ cells) were washed and resuspended in PBS supplemented with 3 % FBS that contained saturating concentrations (1:100 dilution) of the following: FITC-conjugated anti-human monoclonal antibodies (Becton & Dickinson, Mountain View, Calif., USA), anti-CD29-phycoerythrin (PE), anti-CD90-PE, anti-CD34-PE, anti-CD45-PE, anti-CD146-PE or anti-STRO-1-allophycocyanin (APC) for 1 h at room temperature in the dark. As a negative control, PE- and APC-conjugated non-specific mouse IgG1 (Falcon, BD Bioscience, FranklinLakes, N.J., USA) were substituted for the primary antibodies. The cell suspensions were washed twice, resuspended in 3 % FBS/PBS and analysed with a flow cytometry cell sorting Vantage cell sorter (Becton & Dickinson). The data were analysed with a Mod-Fit 2.0 cell cycle analysis program (Becton & Dickinson).

### Statistical analysis

Each experiment was performed at least three times, unless otherwise indicated. Data are reported as the mean ± SE(standard error) deviation from three independent experiments. The significance of the differences between the experimental and the control groups was determined by using one-way analysis of variance; *P* < 0.05 indicated statistical significance.

## Results

### Dental pulp tissue contains the inflammasome components NLRP3, caspase-1 and IL-1β

**Fig. 1:**
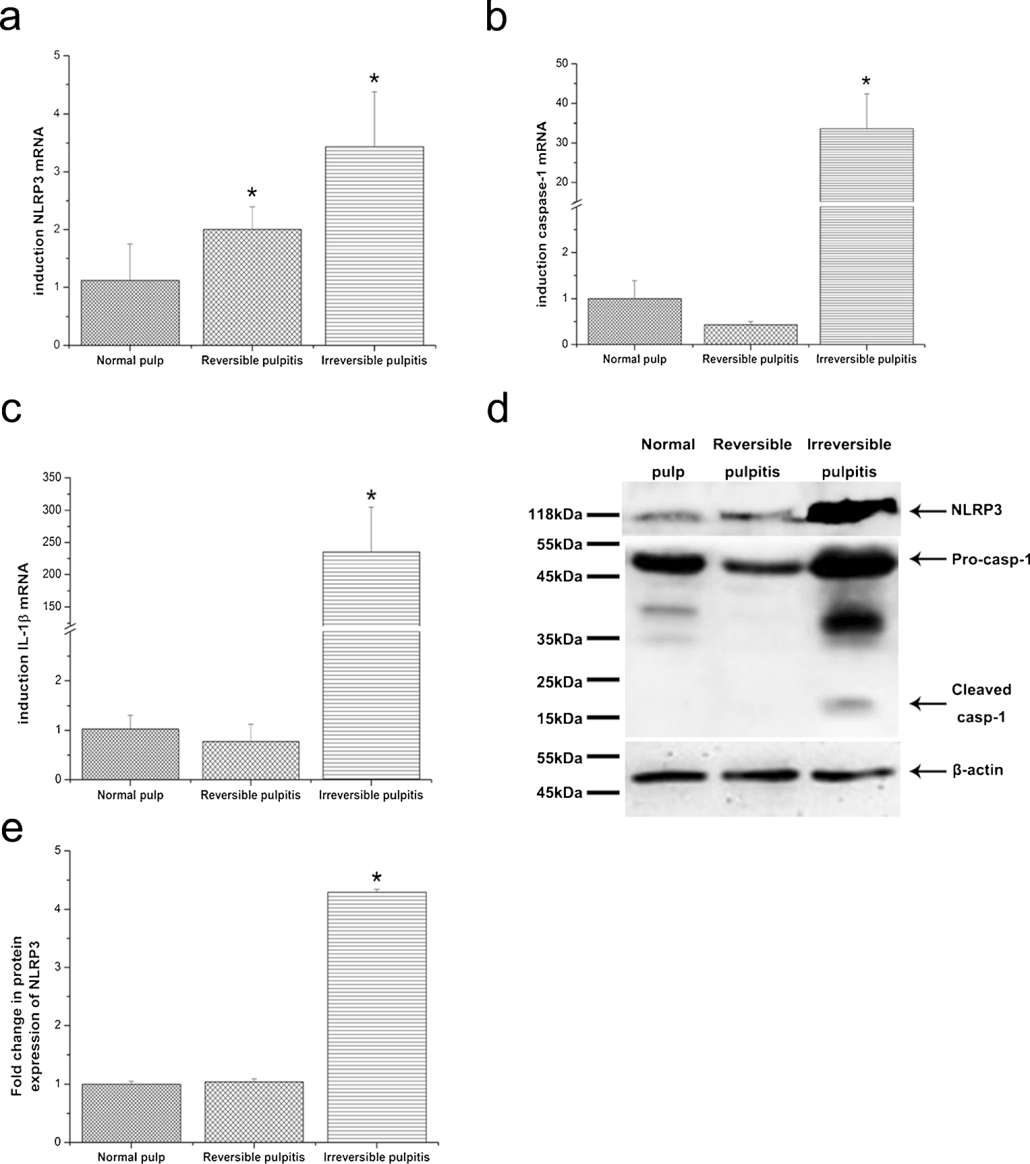
NLRP3 (**a**), caspase-1 (**b**) and IL-1β (**c**) mRNA expression were detected by using quantitative reverse-transcriptase polymerase chain reaction (qRT-PCR; *n* = 3). NLRP3 and caspase-1 protein in dental pulp was detected by Western blotting (*n* = 3; **d**). Blots were stripped and re-probed for β-actin as a loading control. The relative expression intensities were quantified by densitometry (**e**). The level of NLRP3 protein in pulp with irreversible pulpitis was significantly higher than that in the normal and reversible pulpitis groups. Cleaved caspase-1 was only detected in pulp with irreversible pulpitis. Statistical analysis was performed by using one-way analysis of variance (ANOVA). Data are shown as means ± SE (standard error). *P < 0.05 when compared with the other two groups

The expression of NLRP3, caspase-1 and IL-1β was analysed by the detection of the relevant protein and mRNA in normal pulp and in pulp with reversible pulpitis or irreversible pulpitis. As shown in Fig. 1, the expression of NLRP3 (Fig. 1a), caspase-1 (Fig. 1b) and IL-1β (Fig. 1c) mRNA was detected in all three specimens of human dental pulp tissue in each clinical group by using qRT-PCR. The level of NLRP3 mRNA was higher in pulp with reversible or irreversible pulpitis than in normal pulp. A significant difference in the mRNA level of caspase-1 and IL-1β was observed only between normal pulp and pulp with irreversible pulpitis.

The expression of the NLRP3 protein was detected in all pulp tissue specimens by Western blotting. As shown in Fig. 1, NLRP3 was more strongly expressed in pulp with irreversible pulpitis than in normal pulp or pulp with reversible pulpitis (Fig. 1d, e). Caspase-1 activation was assessed by the appearance of caspase-1 p20. The expression of pro-caspase-1 protein was detected in all human dental pulp tissue specimens, whereas the expression of active caspase-1 (p20) was detected only in pulp with irreversible pulpitis (Fig. 1d).

Notably, teeth with reversible pulpitis showed a significantly different NLRP3 mRNA level from that in healthy tissue; however, this difference was not observed at the protein level (Fig. 1d, e).

### Immunofluorescence staining in pulp tissue

**Fig. 2:**
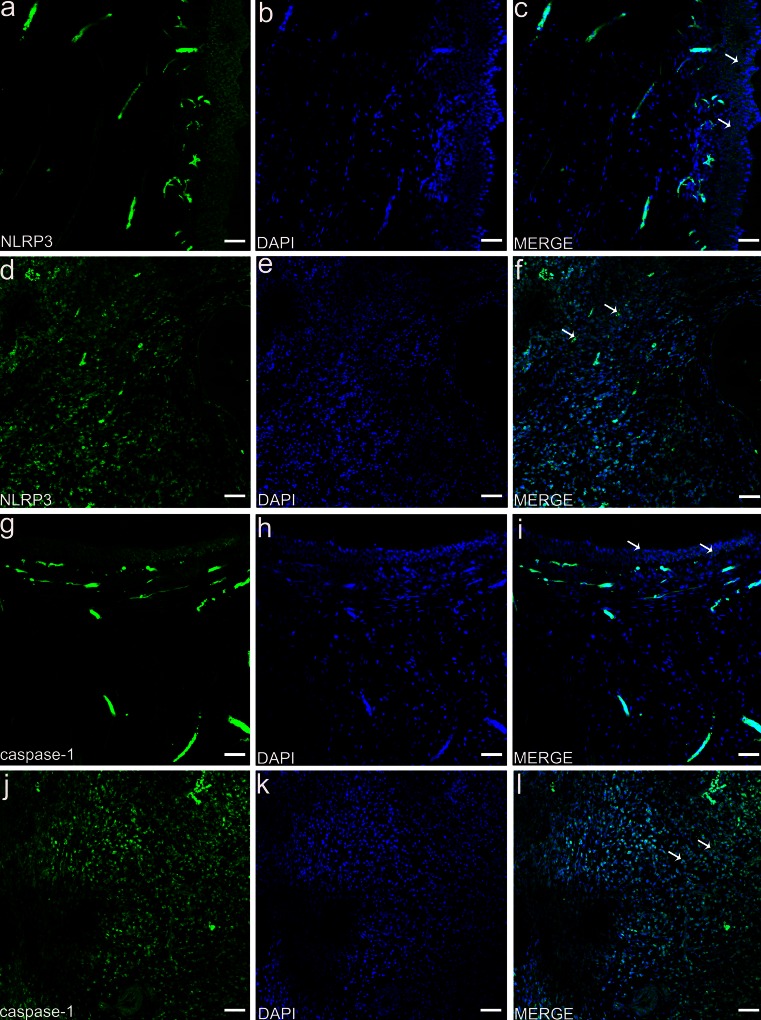
Immunofluorescence staining of NLRP3 and caspase-1 in dental pulp tissue. Two groups were stained for NLRP3 and caspase-1: the normal dental pulp tissue and the irreversible pulpitis tissue. Nulei were stained with 4,6-diamidino-2-phenylindole (*DAPI*). Odontoblast layers (*white arrows*) were positive for NLRP3 (**a–c**) and caspase-1 (**g–i**) in normal dental pulp tissue. Dental pulp cells (*white arrow*) were positive for NLRP3 (**d–f**) and caspase-1 (**j–l**) in the irreversible pulpitis tissue. *Bar* 50 μm

As shown in Fig. 2, the odontoblast layer stained positively for NLRP3 (Fig. 2a-c) and caspase-1 (Fig. 2g-i) in normal pulp tissue. However, in tissue showing irreversible pulpitis, the odontoblast layers were disrupted and dental pulp cells displayed extensive staining for NLRP3 (Fig. 2d-f) and caspase-1 (Fig. 2j-l).

### Isolation and characterization of HDPFs and normal HDPFs containing inflammasome components NLRP3 and caspase-1

**Fig. 3:**
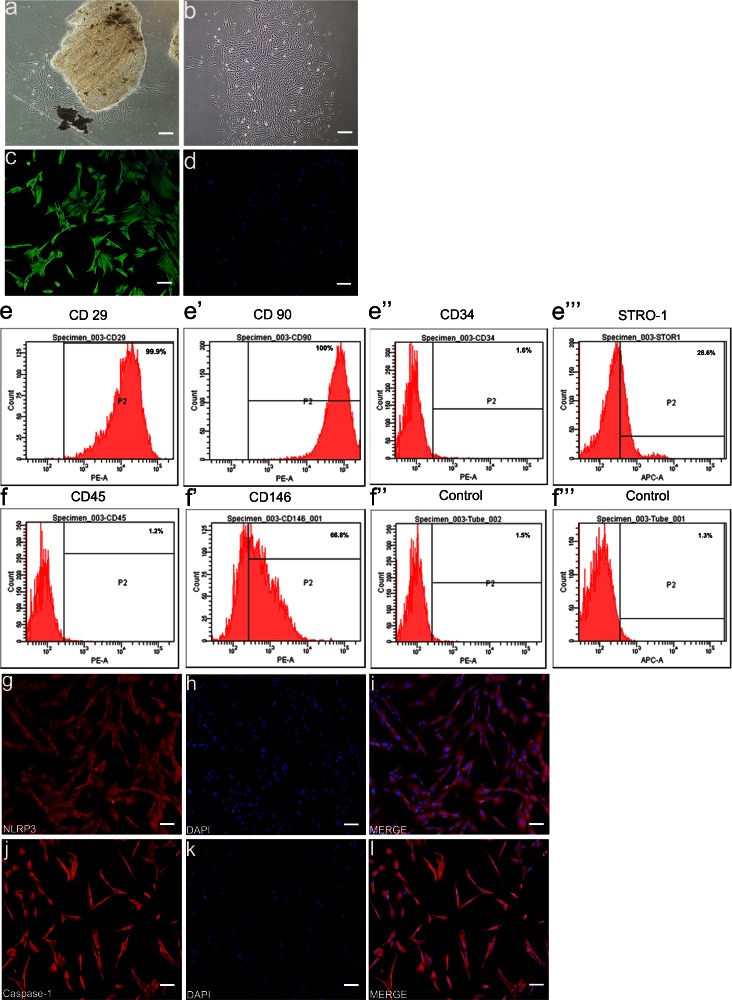
Isolation and characterization of human dental pulp fibroblasts and immunocytochemistry of NLRP3 and caspase-1 in normal human dental pulp fibroblasts. Morphology of primary culture expanded human dental pulp cells at day 5 (**a**) and monoclonal cells at day 15 (**b**). Characterization of HDPFs by immunocytochemical staining: positive immunostaining for vimentin (**c**); negative immunostaining for keratin (**d**). Analysis of molecular surface antigen markers in HDPFs assayed by flow cytometry (*P2* positive zone of antigen) showing that HDPFs were negative for CD34 (**e’’**) and CD45 (**f**), whereas they were positive for CD29 (**e**) and CD90 (**e’**); of the cells, 66.8 % were CD146-positive (**f’**) and 28.6 % STRO-1-positive (**e’’’**). PE- (**f’’**) and APC- (**f’’’**) conjugated non-specific mouse IgG1 served as negative controls. Immunocytochemistry of NLRP3 and caspase-1 in normal HDPFs at the fourth passages showing the presence of NLRP3 (**g–i**) and caspase-1 (**j–l**) in the cytoplasm. Nuclei were stained with DAPI. *Bar* 50 μm

Human dental pulp cells were successfully isolated from the pulp tissue of extracted third molars. On morphological observation, the primary cells presented clone-like growth after they were incubated for 72 h. The cultures were heterogeneous and contained cells that ranged from narrow and spindle-shaped to large and polygonal (Fig. 3a). After the monoclonal cells had been isolated by limiting dilution, they were fibroblast-like or stellate in shape and were more homogeneous in size (Fig. 3b).

HDPFs obtained from the cell clones that were grown for 14 days were characterized by immunocytochemical staining and flow cytometric analysis. Immunocytochemical staining of HDPFs revealed that the cells were positive for vimentin (Fig. 3c) and were negative for keratin (Fig. 3d). Flow cytometric analysis revealed that these cells were negative for CD34 (Fig. 3e’’) and CD45 (Fig. 3f). The cultures contained 99.9 % CD29-positive cells (Fig. 3e), 100 % CD90-positive cells (Fig. 3e’), 66.8 % CD146-positive cells (Fig. 3f’) and 28.6 % STRO-1-positive cells (Fig. 3e’’’).

The expression of NLRP3 and caspase-1 were examined in normal HDPFs by using immunocytochemical staining. We found that the normal HDPFs contained the inflammasome components NLRP3 (Fig. 3g-i) and caspase-1 (Fig. 3j-l) in the cytoplasm.

### ATP plus ultra-pure LPS increases expression of P2X_7_, NLRP3, caspase-1 and IL-1β in HDPFs

**Fig. 4:**
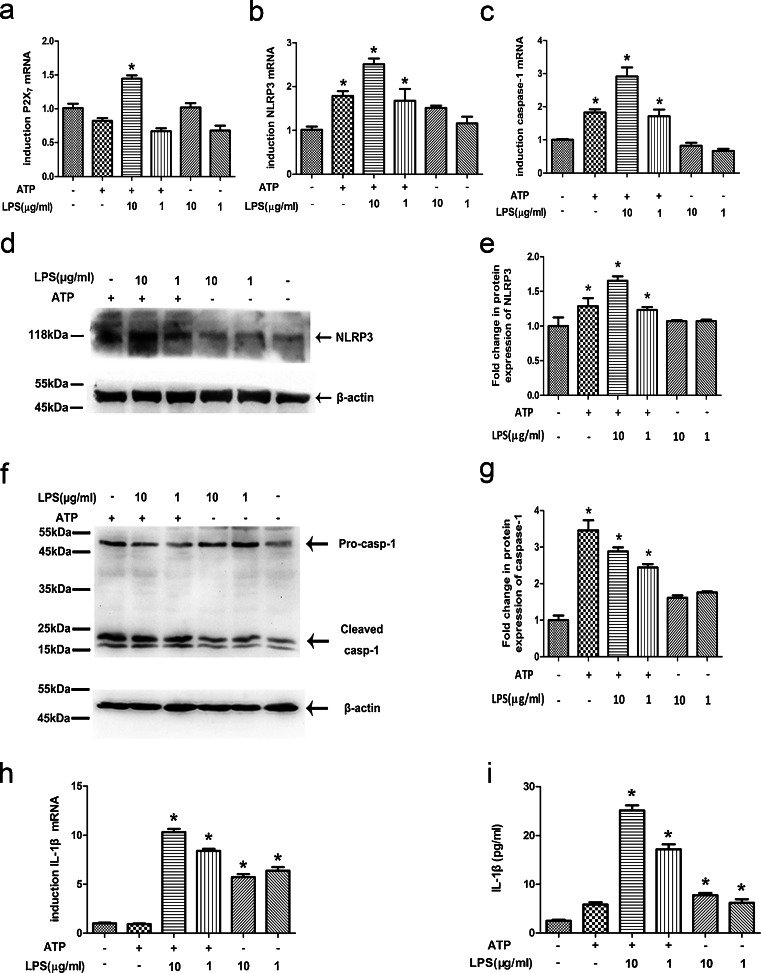
ATP plus lipopolysaccharide (*LPS*) increases the expression of P2X_7_, NLRP3, caspase-1 and IL-1β in human dental pulp fibroblasts. Human dental pulp fibroblasts were incubated with LPS at various concentrations in the presence or absence of ATP (5 mM) stimulation for 2 h. At 6 h post-incubation with LPS, the mRNA expression of P2X_7_ (**a**), NLRP3 (**b**), caspase-1 (**c**) and IL-1β (**h**) in HDPFs was analysed by qRT-PCR, whereas the protein levels of NLRP3 (**d**), caspase-1 (**f**) and IL-1β (**i**) were analysed by Western blotting and ELISA. The relative band intensities were determined by densitometry (**e**, **g**). Statistical analysis was performed by using one-way ANOVA. Data are shown as means ± SE. *P < 0.05 when compared with the untreated control

The expression of P2X_7_, NLRP3, caspase-1 and IL-1β mRNA in HDPFs was examined in response to ATP only, LPS only and ATP plus LPS. The optimal LPS and ATP concentrations needed to elicit a response were also determined. The level of P2X_7_ (Fig. 4a), NLRP3 (Fig. 4b), caspase-1 (Fig. 4c) and IL-1β (Fig. 4h) mRNA was determined by qRT-PCR. P2X_7_, NLRP3, caspase-1 and IL-1β mRNA expression was found to increase markedly after HDPFs had been pulsed with ATP (5 mM) for 2 h followed by LPS exposure (10 μg/ml) for 6 h. The induction of NLRP3, caspase-1 and IL-1β mRNA expression was still detectable when the LPS concentration was 1 μg/ml. Furthermore, ATP treatment alone (5 mM) for 6 h also induced NLRP3 and caspase-1 mRNA expression, albeit to a lesser extent than in the ATP-plus-LPS-treated group; however, ATP alone was not able to induce IL-1β mRNA expression. LPS treatment alone (10 or 1 μg/ml) did not induce NLRP3 and caspase-1 mRNA expression, although it induced IL-1β mRNA expression. The protein levels of NLRP3 (Fig. 4d, e), caspase-1 (Fig. 4f, g) and IL-1β (Fig. 4i) were analysed by Western blotting and ELISA. Caspase-1 activation was assessed by the appearance of caspase-1 p20. Compared with untreated cells, the ATP plus LPS incubation resulted in the activation of NLRP3 and caspase-1 and induced the secretion of IL-1β. In contrast, ATP treatment alone activated NLRP3 and caspase-1 but did not induce IL-1β secretion. LPS treatment alone did not induce the activation of NLRP3 or caspase-1; however, it induced IL-1β secretion.

### Effect of ATP plus ultra-pure LPS over time on expression of P2X_7_, NLRP3, caspase-1 and IL-1β in HDPFs

**Fig. 5:**
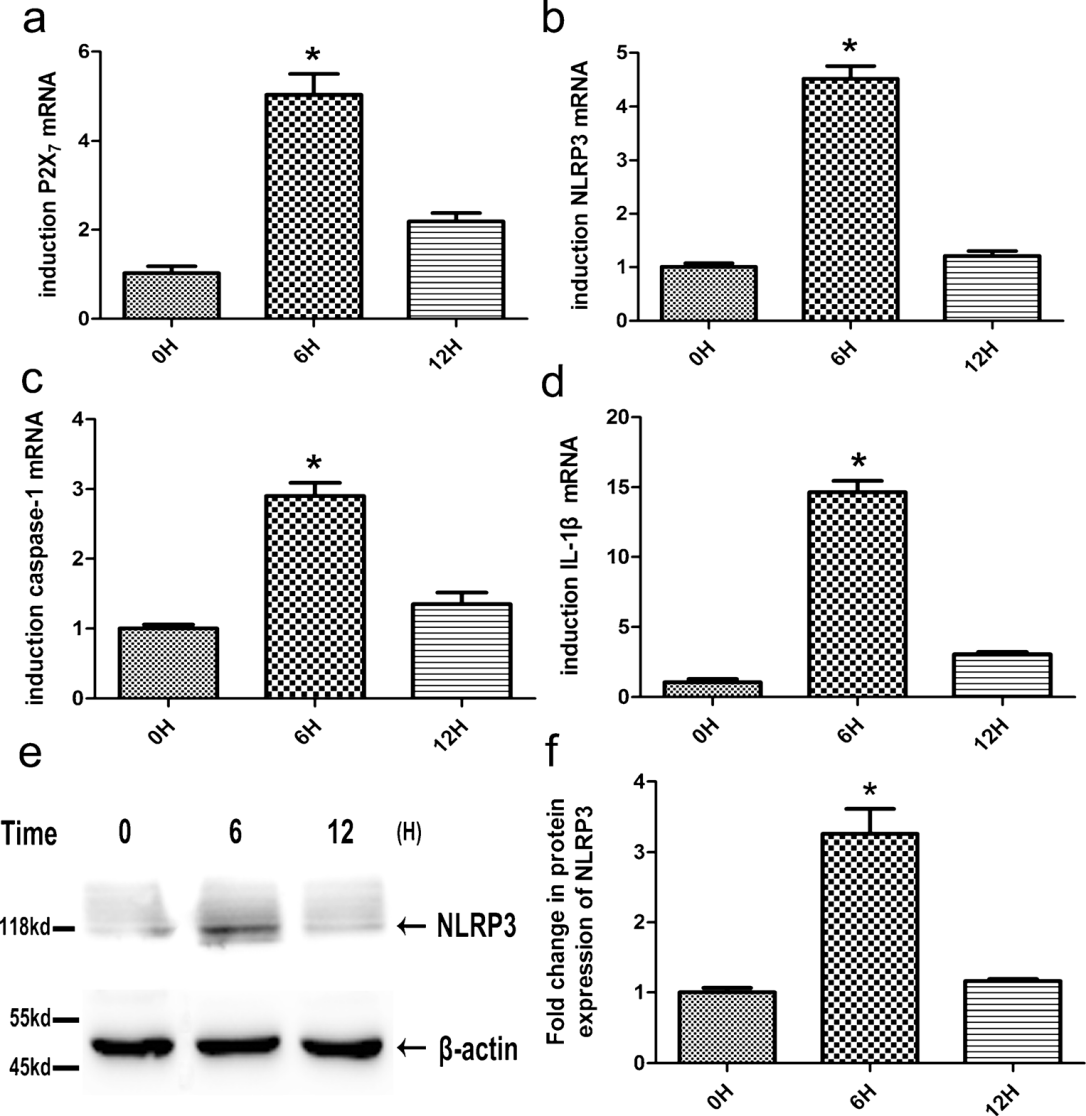
Effect of ATP plus LPS on the expression of P2X_7_, NLRP3, caspase-1 and IL-1β in human dental pulp fibroblasts over time. After an ATP (5 mM) pulse for 2 h, human dental pulp fibroblasts were exposed to LPS (10 μg/ml) for the indicated times (*0H* 0 h, *3H* 3 h, *12H* 12 h). The mRNA expression of P2X_7_, NLRP3, caspase-1 and IL-1β in human dental pulp fibroblasts were analysed by qRT-PCR (**a–d**) and the protein levels of NLRP3 were analysed by Western blotting (**e**). The relative band intensities were determined by densitometry (**f**). Statistical analysis was performed by using one-way ANOVA. Data are shown as means ± SE. *P < 0.05 when compared with the 0H group

A time course to detect P2X_7_ (Fig. 5a), NLRP3 (Fig. 5b), caspase-1 (Fig. 5c) and IL-1β (Fig. 5d) mRNA expression in HDPFs in response to ATP plus ultra-pure LPS was performed. The optimal ATP and LPS concentrations necessary to elicit a response were also determined. After an ATP pulse (5 mM) for 2 h, HDPFs were exposed to LPS (10 μg/ml) for various amounts of time (0, 6 and 12 h) and the levels of P2X_7,_ NLRP3, caspase-1 and IL-1β mRNA were determined by qRT-PCR. The maximal induction of P2X_7_, NLRP3, caspase-1 and IL-1β mRNA expression was observed after 6 h of exposure to 10 μg/ml LPS and 5 mM ATP. The level of NLRP3 protein (Fig. 5e, f) in the ATP-plus-LPS-treated cell culture supernatants was analysed by Western blotting at 0, 6 and 12 h. The increase in NLRP3 was consistent with the pattern of the increase in NLRP3.

### K^+^ efflux is involved in ATP-plus-LPS-induced inflammasome activation in HDPFs

**Fig. 6:**
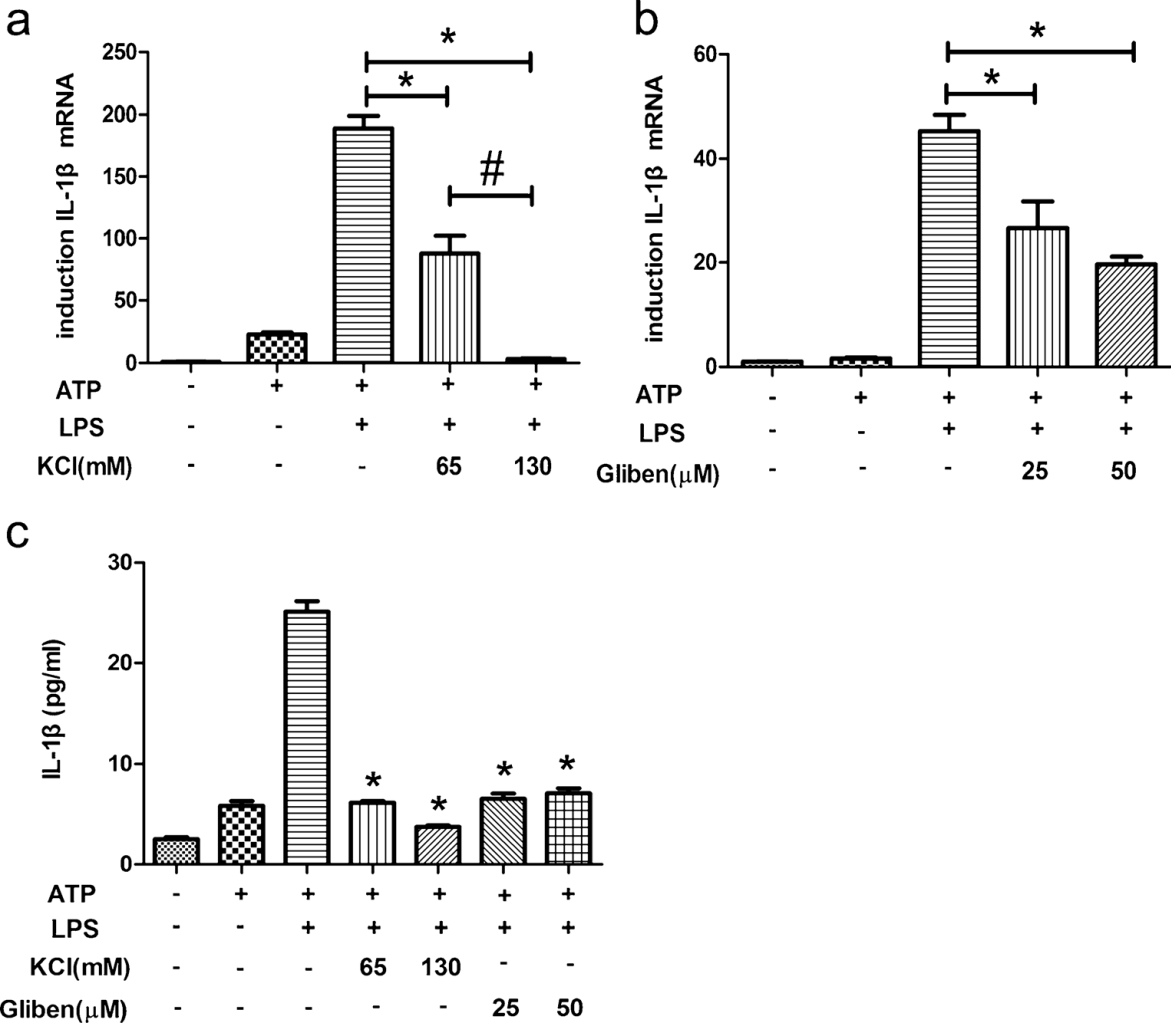
K^+^ efflux is involved in ATP-plus-LPS-induced inflammasome activation in human dental pulp fibroblasts. After an ATP (5 mM) pulse for 2 h, human dental pulp fibroblasts were treated with KCl and glibenclamide (*Gliben*) at the indicated concentrations for 30 min and subsequently incubated with LPS at a concentration of 10 μg/ml. At 6 h post-incubation with LPS, the mRNA expression of IL-1β in human dental pulp fibroblasts was analysed by qRT-PCR (**a**, **b**). At 6 h post-incubation with LPS, the protein level of IL-1β was analysed by ELISA (**c**). Statistical analysis was performed by using one-way ANOVA. Data are shown as means ± SE. *P < 0.05 when compared with the ATP-plus-LPS-treated control, ^#^ *P* < 0.05 when compared with the ATP-plus-LPS- and KCl- (65 mM) treated control

The abundance of IL-1β mRNA and protein was used as an indicator of inflammasome activation. A high extracellular K^+^ concentration (KCl = 130 mM) was used to test whether the inhibition of the K^+^ efflux could influence the ATP-plus-LPS-mediated activation of the NLRP3/caspase-1 inflammasome. After an ATP (5 mM) pulse for 2 h, HDPFs were exposed to a high extracellular K^+^ concentration for 30 min followed by 6 h of LPS (10 μg/ml) induction. Under these conditions, IL-1β mRNA expression was significantly reduced (Fig. 6a). Moreover, when glibenclamide, a selective inhibitor of ATP-dependent K^+^ channels, was used to block K^+^ efflux after a 2-h ATP pulse, the LPS-induced IL-1β mRNA level was significantly reduced (Fig. 6b). The level of IL-1β protein in the LPS-treated cell culture supernatants was analysed by using ELISA at 6 h after LPS exposure; IL-1β protein secretion was reduced under both the extracellular K^+^ condition and the selective K^+^ channel inhibitor condition, which was consistent with the IL-1β mRNA level results (Fig. 6c).

### Reactive oxygen species (ROS) are involved in ATP-plus-LPS-induced inflammasome activation in HDPFs

**Fig. 7:**
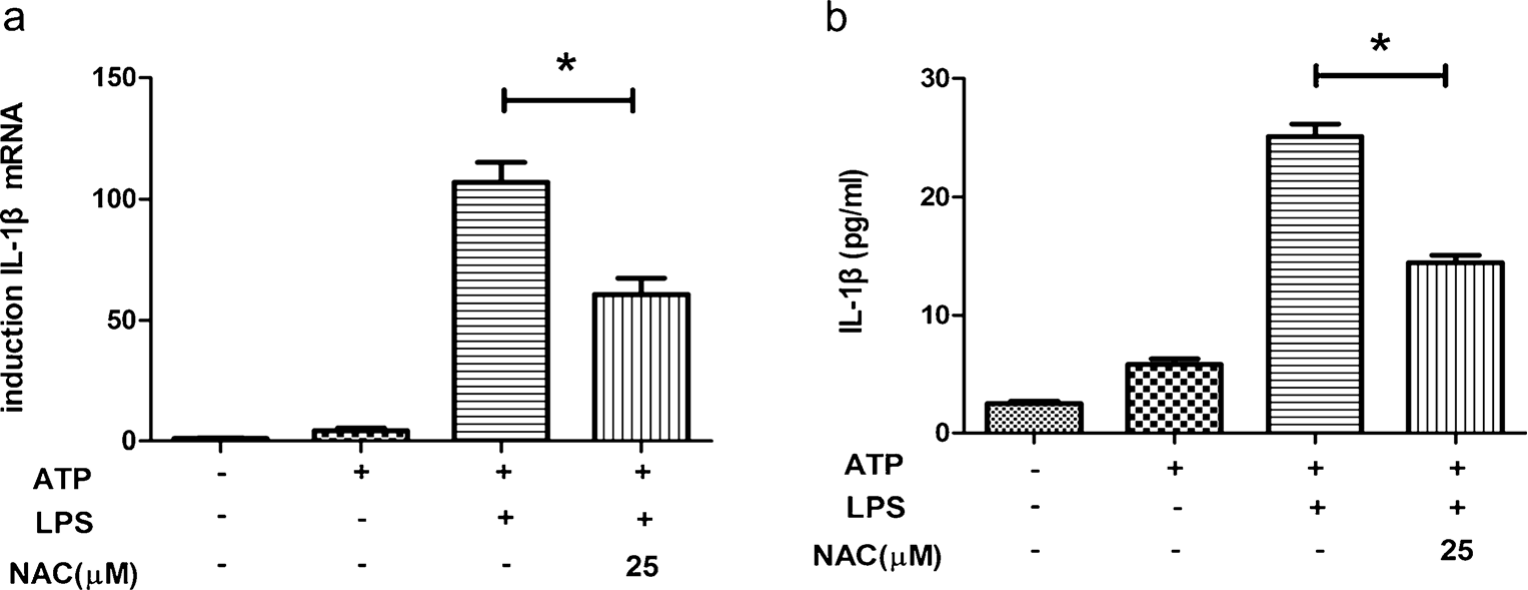
ROS are involved in ATP-plus-LPS-induced inflammasome activation in human dental pulp fibroblasts. After an ATP (5 mM) pulse for 2 h, human dental pulp fibroblasts were treated with N-acetyl cysteine (*NAC*) at the indicated concentrations for 30 min and subsequently incubated with LPS at a concentration of 10 μg/ml. At 12 h post-incubation with LPS, the mRNA and protein levels of IL-1β in human dental pulp fibroblasts were analysed by qRT-PCR (**a**) and ELISA (**b**), respectively. Statistical analysis was performed by using one-way ANOVA. Data are shown as means ± SE. *P < 0.05 when compared with the ATP-plus-LPS-treated control

We used N-acetyl cysteine (NAC), an anti-oxidant that neutralizes ROS, to determine whether ROS were involved in LPS-mediated NLRP3 inflammasome activation. The levels of IL-1β mRNA and protein were used as indicators of inflammasome activation. After an ATP (5 mM) pulse for 2 h, HDPFs were exposed to NAC (25 μM) for 30 min followed by 12 h LPS induction. The results showed that IL-1β secretion (Fig. 7a, b) was significantly reduced upon treatment with 25 μM NAC, suggesting that ROS also contributed to NLRP3/caspase-1 inflammasome activation during ATP plus LPS incubation.

## Discussion

The NLR (NACHT-LRR proteins) family is an important “node” in the innate immune system for processing and transducing inflammatory signals. NLR family activation initiates signal transduction pathways that activate caspase-1 and release pro-inflammatory cytokines, ultimately eliminating microbial invaders (Martinon 2010; Nakanishi et al. 2011). The NLRP3 inflammasome, which comprises the NLRP3 scaffold, the ASC (PYCARD) adaptor and caspase-1, is currently the most fully characterized inflammasome in the NLR family (Hahn et al. 2000; Ogura et al. 2006; Palosaari et al. 2000). Previous studies have demonstrated that most immune cells, including macrophages, monocytes, neutrophils, dendritic cells and B and T lymphocytes, have the NLRP3/caspase-1 inflammasome pathway (Pétrilli et al. 2007; Philpott and Girardin 2004; Qureshi et al. 1999). This inflammasome is a molecular platform that is activated upon cellular infection or stress triggering the maturation of pro-inflammatory cytokines, such as IL-1β and engaging innate immune defences (Hahn et al. 2000; Hirao et al. 2009). Notably, some non-immune cells can also activate inflammasomes. For example, when keratinocytes are exposed to skin irritants or ultraviolet B (UVB) irradiation, the NLRP3 inflammasome is activated (Rathinam et al. 2012; Sahoo et al. 2011). After stimulation, vascular endothelial cells can also express NLRP3, which subsequently induces the activation of the inflammasome pathway and IL-1β secretion (Schroder and Tschopp 2010). Recent studies have demonstrated that the NLRP3 inflammasome can be expressed in normal human dental pulp cells and tissues (Song et al. 2012). Here, we found that the NLRP3/caspase-1 inflammasome pathway can be activated in pulp tissues with irreversible pulpitis. We also studied the specific mechanism of activation of the NLRP3/caspase-1 inflammasome pathway in HDPFs, which are regarded as the main part of human dental pulp cells. These observations indicate that the NLRP3/caspase-1 inflammasome pathway plays an important role in human dental pulp cells and tissues. IL-1β is one of the most powerful pro-inflammatory cytokines and has been shown to be protective in several bacterial, viral and fungal infection models (Staquet et al. 2008). The main function of IL-1β during infection is the induction of several responses, including the rapid recruitment of neutrophils to inflammatory sites, the activation of endothelial adhesion molecules, the induction of cytokines and chemokines, the induction of the febrile response and the stimulation of specific types of adaptive immunity, such as the Th17 response (Staquet et al. 2008). Studies have shown that, in most immune cells (including macrophages and monocytes), IL-1β up-regulation in response to bacteria and bacterial products (such as LPS and lipoteichoic acid) engages innate immune defences.

In this study, we provided evidence of NLRP3 and caspase-1 expression in human dental pulp tissue and HDPFs. We analysed the presence, localization and quantity of NLRP3 and caspase-1 in healthy dental pulp and pulp showing reversible pulpitis or irreversible pulpitis. The mRNA and protein expression of NLRP3 and caspase-1 was observed in all three groups by qRT-PCR and Western blotting. Our data show that NLRP3 gene and protein expression is greater in pulp with irreversible pulpitis than in normal pulp and pulp with reversible pulpitis. The expression of the caspase-1 gene is also increased in pulp showing irreversible pulpitis compared with normal pulp and pulp with reversible pulpitis. These results demonstrate that the NLRP3 inflammasome functions in irreversible pulpitis and that cleaved caspase-1 protein is activated and IL-1β expression is increased in this condition.

Immunofluorescence staining showed that the inflammasome of NLRP3/caspase-1 is present in the odontoblast layers of normal pulp tissues. However, irreversible pulpitis occurs in the pulp tissues when the infection of caries-related microorganisms destroys the odontoblast layers and reaches the dentinal tubules and pulp tissue. The NLRP3 and caspase-1 proteins have been shown to be widely expressed in dental pulp cells. This provides further evidence for the role of the NLRP3/caspase-1 inflammasome in dental pulp cells in mounting a defence against invading foreign bodies.

Gram-negative bacteria have been reported to be closely associated with deep caries and dental pulpitis (Sutterwala et al. 2006). LPS, a major component of the membrane of Gram-negative bacteria, has been shown to be responsible for pulp infection and can trigger a protective inflammatory response in normal pulp (Takahashi et al. 2008; Veerayutthwilai et al. 2007). LPS, which is also called endotoxin, is the most potent immunostimulant of all of the cell-wall components (Nakanishi et al. 2011). It is recognized by a variety of PRRs, including TLR4 (Franchi et al. 2012). Furthermore, many TLR ligands activate the NLRP3 inflammasome, although NLRP3 itself might not directly sense PAMPs. Therefore, in our in vitro study, we selected LPS as a stimulant to probe the NLRP3/caspase-1 inflammasome pathway in HDPFs. We isolated the HDPFs from human dental pulp cells, which are mixed cells and characterized them by using immunocytochemical staining and flow cytometric analysis. HDPFs, which are derived from human dental pulp tissue, belong to mesenchymal cells (Cooper et al. 2010). We revealed that the cells are positive for vimentin, CD29, CD90, CD146 and STRO-1 but negative for keratin, CD34 and CD45, indicating that they are sourced from the mesenchymal cells, not endothelial cells. Subsequently, we showed that the HDPFs, which are non-immune cells, contain the NLRP3/caspase-1 inflammasome and found that the NLRP3/caspase-1 inflammasome pathway can be activated in response to LPS and ATP, leading to the release of pro-inflammatory cytokines, such as IL-1β, to trigger innate immune defences.

A previous study has shown that the robust activation of the NLRP3/caspase-1 inflammasome (and subsequent release of IL-1β) requires the combination of two distinct extracellular triggers. For Signal 1, the innate immune cells, such as macrophages, are stimulated by TLR agonist (such as LPS), which can induce the synthesis of pro-IL-1β. This signal primes the cells. However, pro-IL-1β cleavage and mature cytokine release require a secondary trigger that plays an important role in stimulating caspase-1 activation. TLR priming alone is insufficient to trigger caspase-1 activation. For Signal 2, the trigger can cause ionic perturbations, particularly K^+^ efflux. Purinergic P2X_7_ receptor activated by extracellular ATP is a good example of this signal. Extracellular ATP activates the P2X_7_ ATP-gated ion channel (Kawai and Akira 2011), initiating K^+^ efflux and inducing recruitment of the pannexin-1 membrane pore (Keller et al. 2008). This pore formation allows extracellular NLRP3 agonists to gain access to the cytosol and to directly activate the NLRP3/caspase-1 inflammasome (Keller et al. 2008). In this study, we demonstrated that ATP alone activates the NLRP3/caspase-1 inflammasome in HDPFs but does not induce the release of IL-1β. In contrast, LPS alone does not induce the activation of the NLRP3/caspase-1 inflammasome but induces the release of IL-1β. Moreover, exposure to the combination of ATP and LPS stimulates the purogenic P2X_7_ ATP-gated ion channel, thereby activating the NLRP3/caspase-1 pathway and inducing the maturation and release of the downstream pro-inflammatory factor IL-1β. Based on these results, we hypothesize that treatment with ATP plus LPS promotes pore formation in cells, thereby causing K^+^ efflux and subsequent activation of the NLRP3 inflammasome. Thus, we investigated whether the purogenic P2X_7_ ATP-gated ion channel is involved in LPS-induced IL-1β expression in HDPFs. Our observation that both high extracellular K^+^ levels or a selective K^+^ channel inhibitor significantly suppresses IL-1β production suggests that the purogenic P2X_7_ ATP-gated ion channel is involved in LPS-induced NLRP3 inflammasome activation in HDPFs. In addition, we found that LPS alone stimulates HDPFs to induce the release of IL-1β but not to activate the NLRP3/caspase-1 inflammasome pathway. This result might have been observed because LPS, which is a TLR agonist, causes TLR activation, resulting in the synthesis of the precursor form of the cytokine IL-1β.

Recent findings suggest that ROS, including hydrogen peroxide (H_2_O_2_), the superoxide anion (O_2_) and the hydroxyl radical (OH), are produced in response to NLRP3 activators. ROS are considered to be activators of the NLRP3/caspase-1 pathway in immune cells (Watanabe et al. 2007). The most striking features related to NLRP3 activators are reported to be a K^+^ efflux and a decrease of K^+^ concentration in the cytoplasm (Wilson et al. 1994). Interestingly, K^+^ efflux has been shown to be associated with ROS production at the membrane in plants (Xiang et al. 2011) and the induction of ROS production in human granulocytes (Zhou et al. 2011). In this study, the neutralization of ROS with anti-oxidants significantly diminishes IL-1β secretion in response to ATP plus LPS, suggesting that ROS, at least in part, contributes to LPS-induced NLRP3 inflammasome activation.

**Fig. 8:**
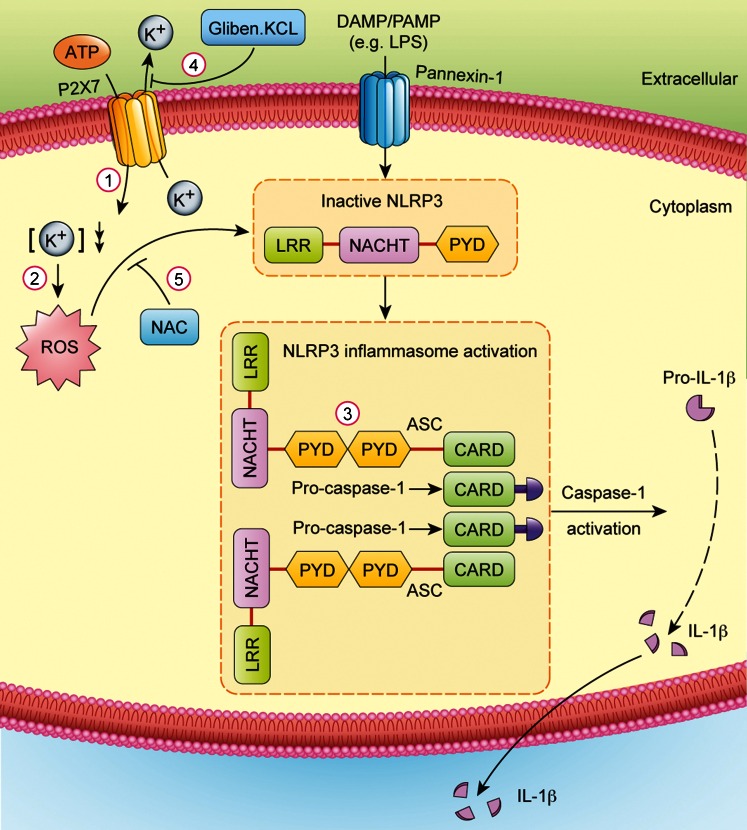
NLRP3/caspase-1 inflammasome pathway in human dental pulp fibroblasts. *1* The NLRP3 agonist ATP stimulates the purogenic P2X_7_ ATP-gated ion channel triggering K^+^ efflux and inducing the formation of the pannexin-1 hemichannel and thus allowing extracellular LPS to enter the cytosol and directly engage NLRP3. *2* The efflux of K^+^ contributes to the decrease in the K^+^ concentration in the cytoplasm. A low intracellular K^+^ concentration has been proposed to trigger ROS generation, which can also activate NLRP3. *3* NLRP3/ASC inflammasome activation results in caspase-1 activation and IL-1β secretion. *4* KCl and gliben were used to block K^+^ efflux and inhibit the activation of NLRP3. *5* NAC was used to block the effect of ROS and inhibit the activation of NLRP3 (*DAMP/PAMP* danger-associated molecular patterns/pathogen-associated molecular patterns, *LRR* leucine-rich repeat, *PYD* pyrin domain, *CARD* caspase recruitment domain)

As summarized in Fig. 8, we speculate that the immune activation of HDPFs involves the activation of the purogenic P2X_7_ ATP-gated ion channel by extracellular ATP and LPS, which in turn triggers K^+^ efflux and induces the gradual recruitment of the pannexin-1 membrane pore. Pore formation then allows extracellular LPS to gain access to the cytosol and directly activate the NLRP3/caspase-1 inflammasome, thereby resulting in the release of the pro-inflammatory cytokine IL-1β. Furthermore, the decrease in the K^+^ concentration in the cytoplasm leads to the generation of ROS. The formation of ROS further activates the NLRP3/caspase-1 pathway, leading to further release of the pro-inflammatory cytokine IL-1β.

In conclusion, this report demonstrates that tissue with irreversible pulpitis contains more functional NLRP3/caspase-1 than healthy pulp and pulp with reversible pulpitis tissue. NLRP3 and caspase-1 are expressed to a different extent in the odontoblast layer in healthy pulp and in inflamed pulp tissue. In inflamed pulpal tissue, dental pulp cells are extensively positive for NLRP3 and caspase-1. We also showed that the NLRP3/caspase-1 inflammasome pathway in HDPFs is activated by LPS through a process involving the ATP-activated P2X_7_ receptor ATP-gated ion channel. The production of ROS also induces the NLRP3 inflammasome. The effect of the activation of the ATP-gated ion channel and the production of ROS is similar. Both processes mediate the activation of the NLRP3/caspase-1 inflammasome pathway, thereby resulting in the release of the proinflammatory cytokine IL-1β, which contributes to the innate immune response.
